# Clinical and event-based outcomes of patients with mucopolysaccharidosis VI receiving enzyme replacement therapy in Turkey: a case series

**DOI:** 10.1186/s13023-021-02060-4

**Published:** 2021-10-19

**Authors:** Aslı İnci, İlyas Okur, Leyla Tümer, Gürsel Biberoğlu, Murat Öktem, Fatih Ezgü

**Affiliations:** Department of Pediatrics, Division of Pediatric Metabolic Diseases, Faculty of Medicine, Gazi University, Mevlana Bulvarı No 29, Emniyet Mahallesi, Yenimahalle, Ankara, 06560 Turkey

**Keywords:** Case series, Galsulfase, Enzyme replacement therapy, Mucopolysaccharidosis VI

## Abstract

**Background:**

The objective of this study was to describe clinical manifestations and events of patients with mucopolysaccharidosis (MPS) VI in Turkey who are treated with galsulfase enzyme replacement therapy (ERT). Clinical data of 14 children with MPS VI who were followed up at the Department of Pediatrics of the Gazi University Faculty of Medicine in Ankara, Turkey were retrospectively collected from the patients’ medical records. Patients were selected based on availability of a pre-ERT baseline and follow-up clinical data for a similar period of time (1.9–3.2 years). Event data (occurrence of acute clinical events, onset of chronic events, surgeries) collected during hospital visits and telemedicine were available for up to 10 years after initiation of ERT (2.5–10 years).

**Results:**

Age at initiation of ERT ranged from 2.8 to 15.8 years (mean age 7.5 years). All patients presented with reduced endurance and skeletal abnormalities (dysostosis multiplex) on radiography. Other common clinical manifestations were cardiac valve disease (N = 13), short stature (N = 11), cranial abnormalities on MRI (N = 10), spinal abnormalities on MRI (N = 7), and mild cognitive impairment (N = 6). School attendance was generally poor, and several patients had urinary incontinence. After 1.9 to 3.2 years of ERT, most patients showed improvements in endurance in the 6-min walk test and 3-min stair climb tests; the frequency of urinary incontinence decreased. ERT did not seem to prevent progression of cardiac valve disease, eye disorders, hearing loss, or bone disease. Long-term event-based data showed a high incidence of respiratory tract infections, adenotonsillectomy/adenoidectomy, reduced sleep quality, sleep apnea, and depression before initiation of ERT. The number of events tended to remain stable or decrease in all patients over 2.5–10 years follow-up. However, the nature of the events shifted over time, with a reduction in the frequency of respiratory tract infections and sleep problems and an increase in ophthalmologic events, ear tube insertions, and depression.

**Conclusions:**

This case series shows the high disease burden of the MPS VI population in Turkey and provides a unique insight into their clinical journey based on real-life clinical and event-based data collected before and after initiation of ERT.

**Supplementary Information:**

The online version contains supplementary material available at 10.1186/s13023-021-02060-4.

## Background

Mucopolysaccharidosis (MPS) VI or Maroteaux-Lamy syndrome (OMIM # 253200) is a rare lysosomal storage disorder caused by deficient *N*-acetylgalactosamine-4-sulfatase (EC 3.1.6.12) activity [1, 2]. The resulting accumulation of glycosaminoglycans (GAGs) in tissues and organs can result in musculoskeletal abnormalities, short stature, coarse facial features, pulmonary, cardiac, and neurological disease (spinal cord compression, carpal tunnel syndrome), hepatosplenomegaly, impaired vision, and hearing loss [1, 3]. Cognitive function is generally preserved [2]. The clinical presentation of MPS VI is a continuum ranging from classical disease with early symptom onset, rapid disease progression, and involvement of multiple organ systems to non-classical disease, with later disease onset and slower symptom progression [4, 5].

International guidelines recommend enzyme replacement therapy (ERT) with galsulfase (Naglazyme®, BioMarin Pharmaceutical Inc., Novato, CA, USA) in patients with MPS VI as soon as possible after a confirmed diagnosis [6]. Galsulfase has been approved by the Food and Drug Administration in 2005 and by the European Medicines Agency in 2006 [1, 7, 8]. The pivotal clinical trials of galsulfase have shown rapid and sustained reductions in urinary GAGs (uGAG) and significant and sustained improvements in endurance in the 6-min walk test (6MWT) and 3-min stair climb test (3MWCT), and in pulmonary function in treated patients, as well as an acceptable safety profile [9–13]. Glasulfase has been available to MPS VI patients in Turkey since 2006.

## Materials and methods

### Objectives and study design

The aim of this case series is to describe clinical manifestations and event-based outcomes over time in patients with MPS VI in Turkey before and after initiation of ERT.

Clinical data of 14 children with an enzymatically confirmed diagnosis of MPS VI who were followed up at the Department of Pediatrics of the Gazi University Faculty of Medicine in Ankara, Turkey were retrospectively collected from the patients’ medical records after ethics committee approval. Patients were selected based on availability of a pre-ERT baseline of clinical data and similar follow-up periods of around 2.5 years for clinical outcomes after initiation of ERT.

Clinical data that were collected during hospital visits included anthropometrics (length/height, weight, and pubertal status), endurance in the 6MWT and 3MSCT, musculoskeletal manifestations on radiography (including bone age, determined by the method of Pyle [14]), cranial and spine magnetic resonance imaging (MRI), echocardiography findings, outcomes of ophthalmological and hearing examinations, psychometric evaluation (Bayley Scales and Kaufman Assessment Battery), concomitant medications, and information on urinary incontinence and missed school days.

In addition, event-based data were collected during hospital visits and via telemedicine (telephone or video calls). The events extracted from patient records were those that had an expected impact on patients’ quality of life, including upper airway events (adenoidectomy and/or tonsillectomy, tracheostomy, and any medication for disease-related upper respiratory tract symptoms), mobility events (symptoms necessitating medication, need for a walker or wheelchair, immobilization), cardiovascular events (symptomatic cardiac failure, cardiovascular system abnormalities necessitating initiation of a medication, symptomatic arrhythmia, symptoms necessitating angiography), pain (any disease-related pain), respiratory events (application of mechanical O_2_ therapy, mechanical ventilation, sleep apnea), recurrent infection episodes, including otitis media (number of events), psychiatric events (e.g. depression), neurologic events (any symptom interfering with daily living, any symptoms necessitating surgery), ophthalmologic events (eyeglasses, cataract or corneal opacity impairing vision, any medication for disease-related symptoms), hearing events (hearing aids, cochlear implants, ventilation tubes), and sleep events (reduced quality/shortened duration of sleep, use of medication/melatonin, sweating, nightmares).

All patients included in the case series provided informed consent to publish clinical data.

### Statistical analysis

All data are summarized descriptively. Z-scores (indicating how many standard deviation [SDs] a value deviates from the mean) were calculated for anthropometric data (length/height and weight) based on standard height-for-age curves in non-affected Turkish children [15].

Event-based data were analyzed by calculating the total number of events documented in blocks of 6 months before and after initiation of ERT. Long-lasting events, such as depression, use of a new medication (not including changes in dosing), leg pain, eyeglasses, or snoring were only counted at the time the event started or recurred.

## Results

### Patient demographics and baseline characteristics

The case series included 14 patients, seven males and seven females, with an enzymatically confirmed diagnosis of MPS VI (Table 1). The patients were from different areas in Turkey and can be considered representative of the Turkish population. Age at initiation of ERT ranged from 2.8 years to 15.8 years, with a mean age of 7.5 (SD 4.1) years (median 6.3 years). Six patients (cases 1–6) were below 5 years of age at treatment initiation. Patients had a classical or intermediate phenotype as determined by disease symptoms starting early in childhood (before 6 years of age) and the rate of the progression of symptoms [4, 5]. Parental consanguinity was reported for ten patients. Parents were either first degree cousins (cases 1, 2, 7, 9, and 11), second degree cousins (cases 6, 8, 10, and 12), or third degree cousins (case 13). Cases 1 and 11 are brothers. Genotype data were available for five patients, with four showing the homozygous mutation c.962T>C and one showing the homozygous mutation c.478C>T. Both mutations have been described previously [16, 17].

**Table 1:** Patient demographics and baseline characteristics

Case	Sex	Consan-guinity	Genetic analysis		Age at diagnosis, years	Age at initiation of ERT, years	Duration of follow-up^a^ for clinical outcomes, years	Duration of follow-up^a^ for event-based outcomes, years	Height^b^, cm	Weight^b^, kg	uGAG^b^, µg/mg creatinine	uGAG age-related reference range, µg/mg creatinine (males + females) [38]
			c.DNA	Protein								
**1**	M	Yes	NA	NA	2.8	2.8	2.8	3.0	90	12.0	123.5	8–90
**2**	M	Yes	c.962T>C homozygous	p.Leu321 Pro	3.8	3.8	2.4	9.0	87	14.5	112.8	8–90
**3**	F	No	NA	NA	4.3	4.3	2.4	2.5	109	15.4	65.2	8–90
**4**	F	No	NA	NA	4.7	4.7	2.2	4.0	106	17.0	132.8	8–90
**5**	F	No	NA	NA	4.8	4.8	1.9	2.5	107	21.0	81.6	8–90
**6**	F	Yes	NA	NA	4.8	4.8	2.3	4.0	90	13.0	154.3	8–90
**7**	M	Yes	c.962T>C homozygous	p.Leu321 Pro	5.0	5.8	2.3	8.5	103	15.8	124.4	8–90
**8**	M	Yes	c.478 C>T homozygous	p.Arg160 Ter	6.0	6.8	2.4	10.0	112	20.0	24.3	8–90
**9**	F	Yes	c.962T>C homozygous	p.Leu321 Pro	7.2	7.8	2.4	9.0	99	15.3	307.7	8–90
**10**	F	Yes	c.962T>C homozygous	p.Leu321 Pro	6.5	7.8	2.5	5.5	96	16.0	128.8	8–90
**11**	M	Yes	NA	NA	7.0	8.5	3.2	3.5	116	25.0	201.1	8–90
**12**	M	Yes	NA	NA	11.0	13.8	2.3	5.0	149	50.0	28.3	5–48
**13**	F	Yes	NA	NA	10.0	13.8	2.4	3.5	145	40.0	63.2	5–48
**14**	M	No	NA	NA	13.0	15.8	2.3	2.5	147	49.0	32.3	5–48

^a^Follow-up since initiation of ERT

^b^At initiation of ERT

NA: not available

Duration of follow-up after initiation of ERT varied between 1.9 and 3.2 years for clinical data collected during hospital visits, with a mean of 2.4 (SD 0.3) years (median 2.4 years). Event-based data collected during visits and via telemedicine were available for 2.5 to 1.0 years before and 2.5 to 10.0 years after ERT start. The discrepancy with the follow-up times of clinical and event data are due to the fact that several patients were followed up in a local hospital and were only in contact with our center via telemedicine for several years. Last available follow-up information is from June 2020. All patients were alive at the time of data collection.

### Glycosaminoglycans

At initiation of ERT, normalized uGAG levels ranged from 24.3 to 307.7 µg/mg creatinine (Table 1). After treatment initiation, levels gradually decreased and remained low during follow-up (Additional file 1: S1). At last follow-up (at 24–72 months after initiation of ERT), uGAG ranged from 1.8 to 47.0 µg/mg creatinine.

### Anthropometrics and pubertal status

**Fig. 1:**
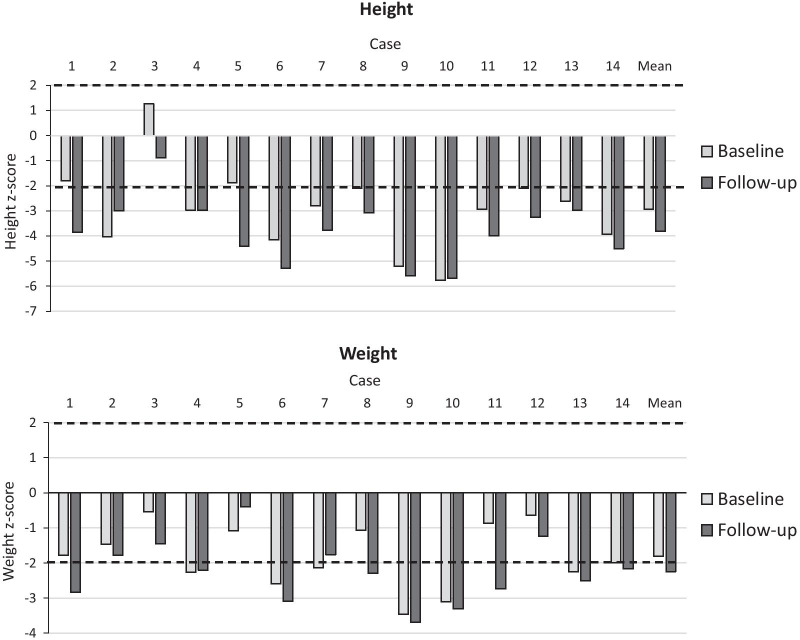
Height and weight z-scores. Height and weight z-scores at initiation of treatment (Baseline) and at last follow-up (1.9–3.2 years). Dashed lines show − 2 and + 2 z-score limits based on standard height-for-age curves in Turkish children [15]

At initiation of ERT all patients > 5 years of age at treatment initiation showed reduced height, i.e. z-scores > 2 SDs from standard height-for-age curves in Turkish children (Fig. 1) [15]. Three of the six patients below 5 years of age at treatment initiation (cases 1, 3, and 5) had baseline heights within normal limits. All 14 patients showed increases in height during follow-up (1.9–3.2 years), with the mean increase being 4.3 (SD 3.7) cm. Mean height z-score changed from − 2.9 (SD 1.7) at treatment initiation to − 3.8 (SD 1.3) at last follow-up. All patients showed a decrease in height z-score over time, except case 2 who showed a slight increase from − 4.0 to − 3.0 and cases 4 and 10 who had stable height z-scores of approximately − 3.0 and − 5.7, respectively (Fig. 1). Only case 3 retained height within 2 SDs from normal values at last follow-up. Comparison of height at treatment initiation and last follow-up with MPS VI-specific growth curves [18] showed a shift towards a higher height percentile in one patient (case 2), no change in six patients (cases 4, 5, 6, 9, 10, 14), and a shift towards a lower percentile in seven patients (cases 1, 3, 7, 8, 11, 12, 13) (Additional file 1: S2).

All patients showed an increase in weight after initiation of ERT. Mean increase in weight was 2.4 (SD 1.5) kg. Mean weight z-score changed from − 1.8 (SD 0.9) at treatment initiation to − 2.2 (SD 0.9) at last follow-up (Fig. 1). Four patients (cases 2, 5, 11, and 12) were overweight at treatment initiation (i.e. body mass index > 85^th^ percentile); at last follow-up only case 5 was still overweight.

All patients had normal pubertal status at baseline and follow-up.

### Endurance

**Fig. 2:**
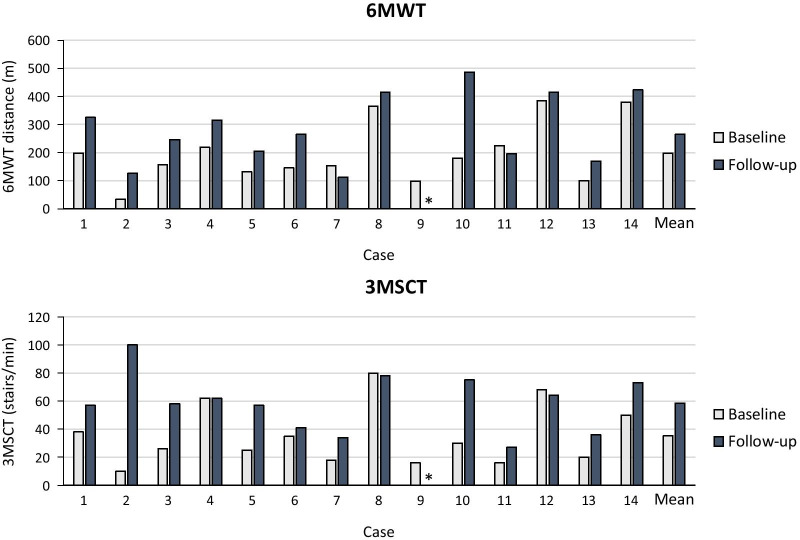
6-min walk test (6MWT) and 3-min stair climb test (3MSCT) outcomes. 6MWT and 3MSCT results before initiation of treatment (Baseline) and at last follow-up. *Case 9 was unable to perform the 6MWT and 3MSCT at follow-up (1.9–3.2 years).

Endurance in the 6MWT at treatment initiation varied considerably between patients (ranging from 34 to 385 m), but all patients had walking distances below those reported for unaffected children of similar age or height [19, 20]. All patients showed increases in walking distance during follow-up (1.9–3.2 years), except cases 7 and 11 (Fig. 2). Case 9 had become immobile at last follow-up, and did not perform the test. The mean increase from pre-treatment baseline to follow-up (excluding case 9) in the 6MWT was 79 (SD 85) m.

There was a great variation in 3MSCT results at treatment initiation between patients (range 3 to 27 stairs/min). All patients showed increases in the number of stairs/min after initiation of ERT, except cases 4, 8 and 12, who showed no change or a minor decrease, and case 9 who became immobile by last follow-up (Fig. 2). Mean increase in the 3MSCT from baseline to follow-up (excluding case 9) was 7.3 (SD 8.4) stairs/min.

### Cardiac manifestations

In all patients, except case 1, echocardiography showed mitral valve disease at initiation of ERT; five patients had aortic valve disease (cases 4, 6, 8, 11, and 13) (Additional file 1: S3). Other findings included tricuspid regurgitation (N = 3), intraventricular septum hypertrophy (N = 2), ventricular extrasystole (N = 1), and minimal pericardial effusion (N = 1).

Valve disease remained absent in case 1 and did not progress during follow-up in cases 6 and 12. Case 13 showed an improvement in mitral regurgitation (Additional file 1: S3). In the other patients, mitral or aortic valve disease progressed. Intraventricular septum hypertrophy was no longer observed at follow-up.

### Ophthalmological manifestations

Ophthalmological abnormalities reported at treatment initiation included mild corneal clouding (cases 2 and 9), iris hypopigmentation and chorioretinal atrophy in case 4, tortuosity of vessels in case 10, and glaucoma in case 13. None of these manifestations resolved during follow-up. Corneal clouding remained present in cases 2 and 9 but did not progress, and newly developed in cases 6, 8, 10 and 13.

### Ear-nose-throat manifestations

At treatment initiation, two patients (cases 4 and 10) showed sensorineural hearing loss. Case 4 had a cochlear implant. During 1.9 to 3.2 years follow-up, sensorineural hearing loss remained present in case 4 but did not worsen, while case 10 developed mixed hearing loss. Three additional patients (cases 6, 9 and 13) developed conductive hearing loss.

### Imaging results

At treatment initiation, all patients showed dysostosis multiplex on radiography. In addition, case 4 showed genu valgum, case 8 showed enlarged ribs, and case 13 showed scoliosis. Bone abnormalities did not resolve during follow-up in any of the patients. Additional abnormalities on radiography, mostly kyphosis or scoliosis, were reported for nine cases (1, 4, 7, 8, 9, 10, 11, 12, and 13) before initiation of ERT and at last follow-up. Bone age remained normal in all patients throughout the study, with deviations from chronological age being smaller than 1.5 years for all cases.

Cranial MRI revealed abnormalities in ten patients (case 2 and cases 6–14), with the most common findings being increased perivascular space, white matter changes, enlarged ventricles, and narrowing of the foramen magnum (Additional file 1: S4). Spinal MRI showed abnormalities in seven patients (cases 2, 7, 8, 10, 12, 13, and 14). The most common findings were narrowing of the spinal canal at the foramen magnum or cervical region and flattening/wedging of vertebral bodies (Additional file 1: S4). Three patients had normal cranial and spinal MRI at treatment initiation (cases 1, 3 and 5); none of them developed abnormalities by last follow-up. Cranial abnormalities did not resolve in any of the patients, and progressed in three patients after treatment initiation (cases 6, 9 and 12). Abnormalities on spinal MRI resolved in two patients (cases 2 and 7) and progressed or newly developed in five patients (cases 6, 8, 9, 11, 12).

### Psychometric evaluation and school attendance

Psychometric evaluation with the Bayley Scales and Kaufman Assessment Battery showed results compatible to age in eight patients (cases 1, 2, 3, 4, 5, 12, 13 and 14) and mild cognitive impairment in six patients (cases 6, 7, 8, 9, 10 and 11) at both treatment initiation and follow-up.

At the time ERT was initiated, the six youngest patients (cases 1–6) did not receive education. The older patients went to school 1 day a week (cases 7, 10, 11, 12, 13, and 14) or 2–3 times a week (cases 8 and 9). Five patients received special education (cases 7–11). By last follow-up, case 4 and 11 started attending school more frequently, while the number of school days dropped to 1 day a week in case 8 and to 0 in case 9.

### Urinary incontinence

Urinary incontinence was commonly reported by patients or their parents. Initially, one patient (case 2) always had urinary incontinence, three patients sometimes (cases 1, 6, and 8), and three rarely (cases 4, 9 and 11) (Additional file 1: S5). After initiation of ERT, the frequency of urinary incontinence increased in cases 9 and 11, decreased in cases 1, 2, 6, and 8, and remained absent in the remaining patients (Additional file 1: S5). At last follow-up, urinary incontinence was absent in nine patients.

### Event-based outcomes

**Fig. 3:**
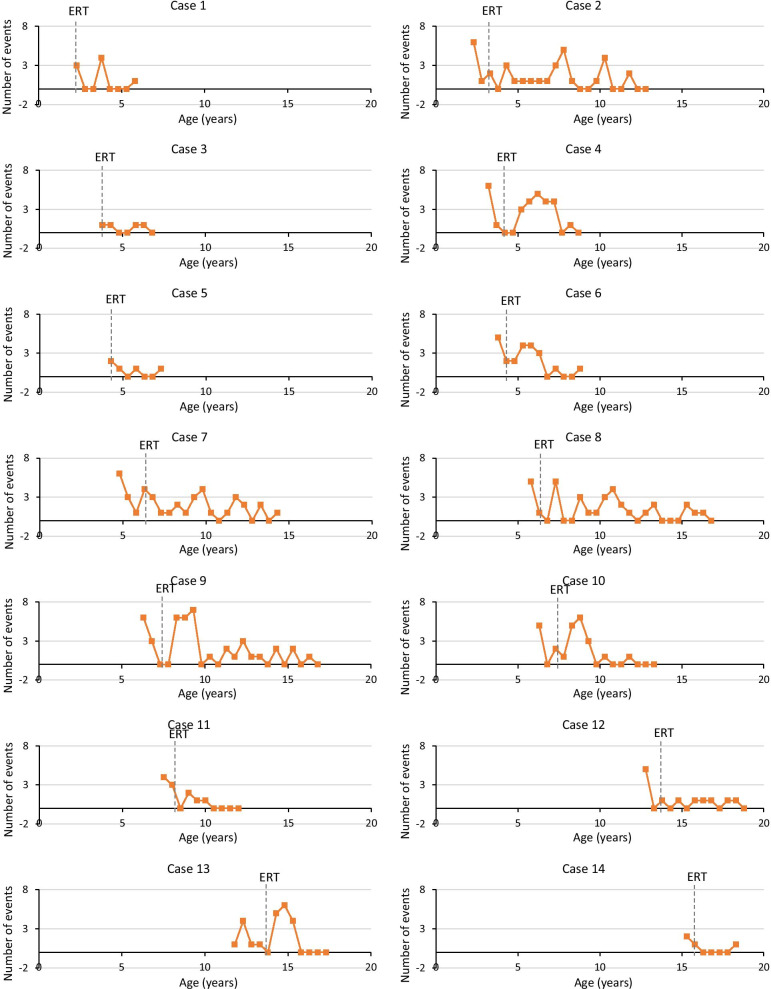
Number of events by age before and after initiation of ERT. Individual patient data. Events include recurrent infection episodes and upper airway/respiratory, mobility, cardiovascular, psychiatric, neurologic, ophthalmologic, hearing, pain, sleep, and abdominal events. Long-lasting events were only counted at the time the event started or restarted after a period of at least 0.5 years. Each data point represents the number of events over a 6-month period before or after initiation of enzyme replacement therapy (ERT)

Long-term disease-related event data were collected during visits and via telemedicine over a period 2.5 to 1.0 years before and 2.5 to 10.0 years after initiation of ERT (Table 1). The number of events before and in the first 6 months after initiation of ERT ranged from 0 to 6. There was a large fluctuation in event numbers over time. However, in the long term, the number of events per 6 months appeared to remain relatively stable or decreased over time in all patients, with none of the patients having more than 1 event in the last 6-month period (Fig. 3 and Additional file 1: S6).

Additional file 1: S7 provides additional information on the nature and incidence of events before and after initiation of ERT. Briefly, the most commonly reported events before initiation of ERT were adenotonsillectomy or adenoidectomy (N = 13 patients), severe upper and lower respiratory tract infections (N = 11), reduced quality of sleep (interrupted sleep, daytime sleepiness, sweating; N = 10), sleep apnea (N = 7) and depression (N = 6). After initiation of ERT, the most striking change was a drop in the frequency of severe respiratory tract infections, with the majority of patients (N = 12) reporting no infections in the last 6 months of follow-up. Sleep problems resolved in seven of ten patients with this finding at treatment initiation. Events that increased in frequency after initiation of ERT are wearing eyeglasses (from 3 before to 12 after initiation of ERT), ear tube insertions (from 0 to 5), use of angiotensin-converting-enzyme ACE) inhibitors (from 1 to 8) and, to a lesser extent, depression (from 7 to 10). Sleep apnea resolved in three of six patients and newly developed in five patients.

## Discussion

The present case series provides information on the clinical presentation of children (2.8 to 15.8 years at treatment initiation) diagnosed with MPS VI in Turkey and contributes to a better insight into their clinical journey while they are treated with ERT.

All 14 patients presented with several, often severe, clinical manifestations of MPS VI at the time ERT was started. They all had reduced height (> 2 SDs below standard height-for-age curves in Turkish children at initiation of ERT [15]), except three of the six patients below 5 years of age at baseline. In addition, all patients had reduced endurance in the 6MWT and skeletal abnormalities (dysostosis multiplex) on radiography, and the majority had cardiac valve disease (mostly involving the mitral valve) and ear-nose-throat or respiratory problems (sleep apnea, respiratory tract infections, hearing loss), and reduced sleep quality. Cranial and spinal MRI mainly revealed abnormalities in the older patients (> 5 years of age), with the most prevalent findings being increased perivascular space, white matter changes, enlarged ventricles, narrowing of the spinal canal at the foramen magnum or cervical region, and flattening/wedging of vertebral bodies. All these clinical manifestations have previously been described in literature [18, 21–26]. None of the patients had abnormal bone age.

Neurocognitive examinations revealed mild cognitive impairment in six patients. The latter is remarkable since MPS VI is generally considered not to affect cognition, despite morphological changes in the brain [25, 26]. A study by Ebbink et al. in 11 children with MPS VI suggested that cognitive development in these patients is mostly determined by familial and social background factors, although the disease can also have an impact on cognitive function in patients with a severe phenotype [27]. In some patients in this case series, visual or hearing deficits may have influenced the test results. In addition, some of our patients may have multiple conditions due to consanguinity of parents. MPS VI also had a big impact on education: none of the patients went to school full-time. Most patients > 5 years of age attended school only 1 day a week. This may also partly explain low scores in cognitive tests for some of the patients. Another finding was that several patients had urinary incontinence. This may be (partly) related to the patients’ young age (for cases 1 and 2) or cognitive decline (cases 6, 8, 9, and 11). Neurogenic bladder, secondary to spinal cord injury, may also have caused incontinence in some of the patients, as has been described for other types of MPS [28].

After receiving ERT for 1.9 to 3.2 years, most patients showed clear improvements in the endurance/mobility tests. The mean increases of 79 m in the 6MWT and 7.3 stairs/min in the 3MSCT are comparable to those seen in the phase 3 study after 2 years follow-up (+ 80 m and + 13.1 stairs/min in those treated continuously with ERT [rhASB/rhASB group]; N = 19) [9]. Of note, in three of four patients that did not show improvements in the 3MSCT tests (cases 4, 8, and 12) endurance was only slightly reduced at baseline (around 400 m), with likely little room for improvement. The increase in endurance seen in our patients and the clinical trials is clinically relevant, since 6MWT distance has been shown to be an independent predictor of morbidity and mortality in patients with cardiac and pulmonary diseases [29, 30]. In addition, four patients showed improvements in urinary incontinence after initiation of ERT. Since this was mostly reported for the younger patients, it could partly be due to ageing. The increased frequency of urinary incontinence observed in two patients (cases 9 and 11) may be due to worsening neurogenic bladder since both patients showed progression of spine abnormalities. Case 9 also showed obvious spinal cord compression.

Height increased in all patients during follow-up. The most favorable growth outcome was seen in case 2, who showed an increase in z-score from − 4.0 at baseline to − 3.0 after 2.4 years of treatment, as well as a shift towards a higher MPS VI-specific height percentile [18]. All other patients remained around the same MPS VI-specific height percentile or shifted towards a lower percentile after initiation of treatment, suggesting no impact of treatment on growth. Despite a decrease in height z-score, height in Case 3 remained within normal limits (z-score within 2 SDs from standard growth curves) at last follow-up.

ERT did not seem to prevent progression of cardiac valve disease, ophthalmological abnormalities, hearing loss, or bone disease in most patients, as could be expected based on current literature [31, 32]. Progressive narrowing of the foramen magnum resulted in spinal cord compression in two patients. These findings underline the importance of regular cardiac, ophthalmological, audiological, and neurological examinations in these patients as stated in international recommendations for the management of MPS VI [6]. The reason for the lack of impact of ERT on these clinical manifestations is still unclear, but has been suggested to be related to poor penetration of the enzyme in the specific tissues [32]. It should be noted that valve disease did not develop in the youngest patient, who was the only patient not affected at initiation of ERT. Although it is possible that this patient had a particular form of the disease not including valve abnormalities, the possibility that ERT prevented the development of valve disease might be considered. Several sibling studies have suggested that ERT may slow down development and progression of valve disease when started early in life [33–35]. Although cardiac valve disease, mostly involving the mitral and aortic valves, continued to progress, intraventricular septum hypertrophy resolved in the two patients with this diagnosis at baseline. The latter finding is consistent with previous studies suggesting that ERT improves or arrests progression of left ventricular remodeling and hypertrophy [22].

The event-based data showed a stabilization or reduction in the number of disease-related events after initiation of ERT in all patients in the long-term (over 2.5 to 10 years follow-up), with none of the patients having more than one event in the last assessed 6-month period. Each of the documented events may interfere with the patients’ daily functioning or quality of life. It should be noted that several patients showed an increase in event numbers in the first period after starting ERT, followed by a reduction. This initial increase may be related to increased monitoring and evaluations after initiation of ERT, which may have resulted in the detection of previously unnoticed clinical manifestations. There appeared to be a shift in the nature of events over time: whereas the frequency of respiratory tract infections and sleep events (reduced sleep quality) decreased markedly over time, other events such as ophthalmologic events (mainly wearing eyeglasses), ear tube insertions, use of ACE inhibitors, and depression became more frequent. ERT also did not seem to prevent new onset of sleep apnea in several patients (although it resolved in three patients).

Whereas uGAG levels appeared to show some relationship with the clinical presentation of patients, they did not predict the disease course after initiation of ERT. Overall, patients with lower uGAG levels (< 100 µg/mg) before initiation of ERT tended to present with less severe impairments in growth and mobility and lower event numbers than those with higher levels, confirming previous findings [36, 37], and none of them showed cognitive impairment. However, cardiac and skeletal abnormalities, as well as abnormalities on cranial and spinal MRI were observed regardless of baseline uGAG levels. Despite differences in phenotypes, endurance tended to improve or stabilize in most patients after treatment initiation and all patients showed a decrease or stabilization in event frequency in the long term, highlighting the importance of treatment of patients with MPS VI across the disease spectrum.

The present study is subject to some limitations inherent to its retrospective nature, including differences in time windows of follow-up visits and varying follow-up periods. Some additional limitations should be acknowledged for the event analysis. Since each event received the same weight in the analysis (i.e. was counted only once), recurrent events such as respiratory tract infections and pain events had a greater impact on total event numbers and changes over time than one-off events such as starting wearing eyeglasses. No distinction was made between events based on severity since the impact of the same event on quality of life can differ significantly between individuals or may change when a patient ages. Also, the data should be interpreted taking into account the variation in the patients’ age at initiation of ERT (with older patients having a bigger risk of having events such as adenotonsillectomy or starting to wear eye glasses before ERT). Additionally, due to the lack of comparable long-term event data for untreated MPS VI patients, it is not possible to determine the impact of ERT on these events. Nonetheless, given the progressive nature of MPS VI, an increase rather than a reduction or stabilization in event rate over time could likely be expected in the absence of treatment. Finally, patient care and interventions other than ERT or aging may also have influenced the frequency of certain events. For example, ear tube insertions (reported for five patients after initiation of ERT) and aging may have contributed to the reduction in frequency of respiratory tract infections.

## Conclusions

This case series provides a unique insight into the clinical presentation and clinical course of patients with MPS VI in Turkey starting ERT during childhood or adolescence. The clinical characteristics and event history at the time of treatment initiation show a high disease burden in this population, including a remarkably high prevalence of mild cognitive impairment and a high impact on education. Overall, longitudinal data after initiation of ERT showed improvements in endurance regardless of age at treatment initiation, confirming clinical trial results [9]. The frequency of disease-related events that may impact on daily life also remained stable or decreased over time in all patients. ERT did not seem to prevent progression of cardiac valve disease, eye disorders, hearing loss or bone disease, underscoring the importance of continued regulatory monitoring of these manifestations [6].

## Supplementary Information

**Additional file 1.** **S1**. Urinary glycosaminoglycan (uGAG) levels over time. **S2**. Height and mucopolysaccharidosis VI-specific height percentile (based on Quartel et al., 2015 [1]) before treatment initiation and follow-up (boys and girls). **S3**. Cardiac echocardiography findings at initiation of enzyme replacement therapy (baseline) and at last follow-up. **S4**. Cranial and spinal magnetic resonance (MRI) outcomes at initiation of enzyme replacement therapy at baseline (BL) and follow-up (FU). **S5**. Urinary incontinence at initiation of enzyme replacement therapy (baseline) and at last follow-up. **S6**. Cumulative number of events by age before and after initiation of ERT. Individual patient data. **S7**. Summary of even-based outcomes.

## Data Availability

The datasets used and/or analyzed during the current study are available from the corresponding author on reasonable request.

## Abbreviations

3MSCT: 3-Minute stair climb test
6MWT: 6-Minute walk test
ACE: Angiotensin-converting enzyme
ERT: Enzyme replacement therapy
GAGs: Glycosaminoglycans
MPS: Mucopolysaccharidosis
MRI: Magnetic resonance imaging
