# Supplementary material for: Clinical and event-based outcomes of patients with mucopolysaccharidosis VI receiving enzyme replacement therapy in Turkey: a case series
Source: Orphanet J Rare Dis. 2021 Oct 19;16:438. doi: 10.1186/s13023-021-02060-4 (PMC8524901; doi:10.1186/s13023-021-02060-4)
Supplement: Supplementary file 1 — Additional file 1. S1. Urinary glycosaminoglycan (uGAG) levels over time. S2. Height and mucopolysaccharidosis VI-specific height percentile (based on Quartel et al., 2015 [1]) before treatment initiation and follow-up (boys and girls). S3. Cardiac echocardiography findings at initiation of enzyme replacement therapy (baseline) and at last follow-up. S4. Cranial and spinal magnetic resonance (MRI) outcomes at initiation of enzyme replacement therapy at baseline (BL) and follow-up (FU). S5. Urinary incontinence at initiation of enzyme replacement therapy (baseline) and at last follow-up. S6. Cumulative number of events by age before and after initiation of ERT. Individual patient data. S7. Summary of even-based outcomes. [file 13023_2021_2060_MOESM1_ESM.pdf]

Additional file

S1 Urinary glycosaminoglycan (uGAG) levels over time

Patient	uGAG (mg/g creatinine)									
	Baseline	Months after initiation of ERT								
		6	12	18	24	30	36	48	54	72
1	123.5		74.0		5.0					
2	112.8	98.3	38.0		7.7		19.3			12.4
3	65.2	29.0	27.9	7.3	1.8					
4	132.8	112.5	32.5	24.0	12.6	6.7	8.9			
5	81.6	24.1	10.8	28.3	31.5					
6	154.3	98.6	76.8	39.3	11.7	9.6	10.3			
7	124.4	111.6	83.6	56.0				32.0	14.7	
8	24.3	8.4	27.0	25.0	7.0	6.7	1.8			2.0
9	307.7	86.0	224.0	42.0	32.0	115.7	21.0			16.0
10	128.8	78.6		54.7	32.6		42.5		21.7	
11	201.1		120.0		47.0					
12	28.3				11.5		15.5	9.7		
13	63.2	38.0	29.1		29.0		17.4			
14	32.3			4.2	2.2					

S2 Height and mucopolysaccharidosis VI-specific height percentile (based on Quartel et al, 2015 [1]) before treatment initiation and follow-up (boys and girls)

**Case**	**Height at initiation of ERT**		**Height at last follow-up**	
	**Height, cm**	**Percentile**	**Height, cm**	**Percentile**
**1**	90.00	P75-90	94.00	P25-50
**2**	87.00	P25-50	102.00	P50-75
**3**	109.30	>P95	112.00	P90-95
**4**	96.00	P50-75	104.00	P50-75
**5**	97.00	P50-75	99.00	P50-75
**6**	90.00	P25-50	94.80	P25-50
**7**	103.00	P75-90	107.00	P50-75
**8**	112.00	P90-95	115.00	P75-90
**9**	99.00	P25-50	102.00	P25-50
**10**	96.00	P25-50	102.00	P25-50
**11**	116.00	P75-90	120.00	P50-75
**12**	149.00	>P95	152.00	P75-90
**13**	145.00	P90-95	145.00	P75-90
**14**	147.40	P75-90	148.40	(P75-90)[a]

[a]Data for 18 years of age (patients was 18.2 years of age, which is outside the reference curve)

1. Quartel A, Hendriksz CJ, Parini R, Graham S, Lin P, Harmatz P. Growth charts for individuals with mucopolysaccharidosis VI (Maroteaux-Lamy Syndrome). JIMD Rep. 2015;18:1-11.

S3 Cardiac echocardiography findings at initiation of enzyme replacement therapy (baseline) and at last follow-up. AR: aortic regurgitation, MR: mitral regurgitation, TR: tricuspid regurgitation. Green indicates normal mitral/aortic valves, orange indicates abnormal valves

Case	Mitral valve		Aortic valve		Other findings	
	Baseline	Follow-up	Baseline	Follow-up	Baseline	Follow-up
1	Normal	Normal	Normal	Normal	-	-
2	2nd degree MR	2nd degree MR	Normal	2nd degree AR	Minimal TR, ventricular extrasystole	-
3	Mild thickening	Mild thickening	Normal	Mild thickening	-	-
4	2nd degree MR	2nd degree MR	Minimal AR	2nd degree AR	-	-
5	Mild thickening	2nd degree MR	Normal	Normal	-	-
6	2nd degree MR	2nd degree MR	Minimal AR	Minimal AR	Minimal TR	Minimal TR
7	Mild MR	2nd degree MR	Normal	Mild AR	Intraventricular septum hypertrophy	Mild TR
8	2nd degree MR	2nd degree MR	1st degree AR	2nd degree AR	-	-
9	Minimal MR	2nd degree MR	Normal	3rd degree AR	Intraventricular septum hypertrophy, minimal TR	Minimal TR
10	1st degree MR	2nd degree MR	Normal	Normal	-	-
11	2nd degree MR	2nd degree MR	2nd degree AR	3rd degree AR	-	-
12	2nd degree MR	2nd degree MR	Normal	Normal	-	-
13	3rd degree MR	2nd degree MR	2nd degree AR	2nd degree AR	Minimal pericardial effusion	-
14	2nd degree MR	2nd degree MR	Normal	2nd degree AR	_	_

S4 Cranial and spinal magnetic resonance (MRI) outcomes at initiation of enzyme replacement therapy at baseline (BL) and follow-up (FU).

NA: not available; WM: white matter

Case		Cranial MRI	Spinal MRI	Spinal cord compression
**1**	BL	-	-	-
	FU	-	-	-
**2**	BL	Increased perivascular space, WM changes	Mild cervical flattening and narrowing	-
	FU	No change	-	-
**3**	BL	-	-	-
	FU	-	-	-
**4**	BL	-	-	-
	FU	NA[a]	NA[a]	-
**5**	BL	-	-	-
	FU	-	-	-
**6**	BL	Increased perivascular space	-	-
	FU	Increased perivascular space, narrow foramen magnum	Narrow foramen magnum and C1	Mild compression
**7**	BL	Increased perivascular space	Mild cervical flattening	-
	FU	Increased perivascular space	-	-
**8**	BL	Asymmetric enlargement of lateral ventricles	Flattening of vertebral bodies, wedging of cervical vertebrae	-
	FU	Asymmetric enlargement of lateral ventricles	Kyphosis, schmorl nodules at vertebral corpus end plate, flattened vertebrae	-
**9**	BL	Arachnoid cyst	-	-
	FU	Arachnoid cyst, enlarged lateral & 3rd ventricle, WM edema	Narrowing of spinal canal at foramen magnum and C1	Obvious compression
**10**	BL	Increased periventricular space, enlarged ventricles, narrow foramen magnum	Narrow foramen magnum	No symptoms
	FU	Increased periventricular space, enlarged ventricles, narrow foramen magnum, ventriculoperitoneal shunt	Narrow foramen magnum	No symptoms
**11**	BL	Periventricular leukodystrophy	-	-
	FU	Periventricular leukodystrophy	Mild narrowing of foramen magnum	-
**12**	BL	Signal increase in occipital in calvarial bones	Narrow spinal canal at C1-C2	No symptoms

	FU	Prominence in the prepontine system, signal increase in occipital calvarial bones	Narrow spinal canal at C1-C7	No symptoms
**13**	BL	Tetraventricular hydrocephaly, increased periventricular space, narrow foramen magnum	Narrow foramen magnum	No symptoms
	FU	Tetraventricular hydrocephaly, increased periventricular space, narrow foramen magnum, flattened C1-C2	Narrow foramen magnum	No symptoms
**14**	BL	Widening of Virchow Robin space	Narrow spinal canal	No symptoms
	FU	Widening of Virchow Robin space	Narrow spinal canal	No symptoms

[a]MRI could not be performed because of cochlear implants

S5 Urinary incontinence at initiation of enzyme replacement therapy (baseline) and at last follow-up

Case	Age at initiation of ERT	Urinary incontinence	
		**Baseline**	**Follow-up**
**1**	2.8	Sometimes	Never
**2**	3.8	Always	Rarely
**3**	4.3	Never	Never
**4**	4.7	Rarely	Rarely
**5**	4.8	Never	Never
**6**	4.8	Sometimes	Rarely
**7**	5.8	Never	Never
**8**	6.8	Sometimes	Never
**9**	7.8	Rarely	Sometimes
**10**	7.8	Never	Never
**11**	8.5	Rarely	Always
**12**	13.8	Never	Never
**13**	13.8	Never	Never
**14**	15.8	Never	Never

S6 Cumulative number of events by age before and after initiation of ERT. Individual patient data

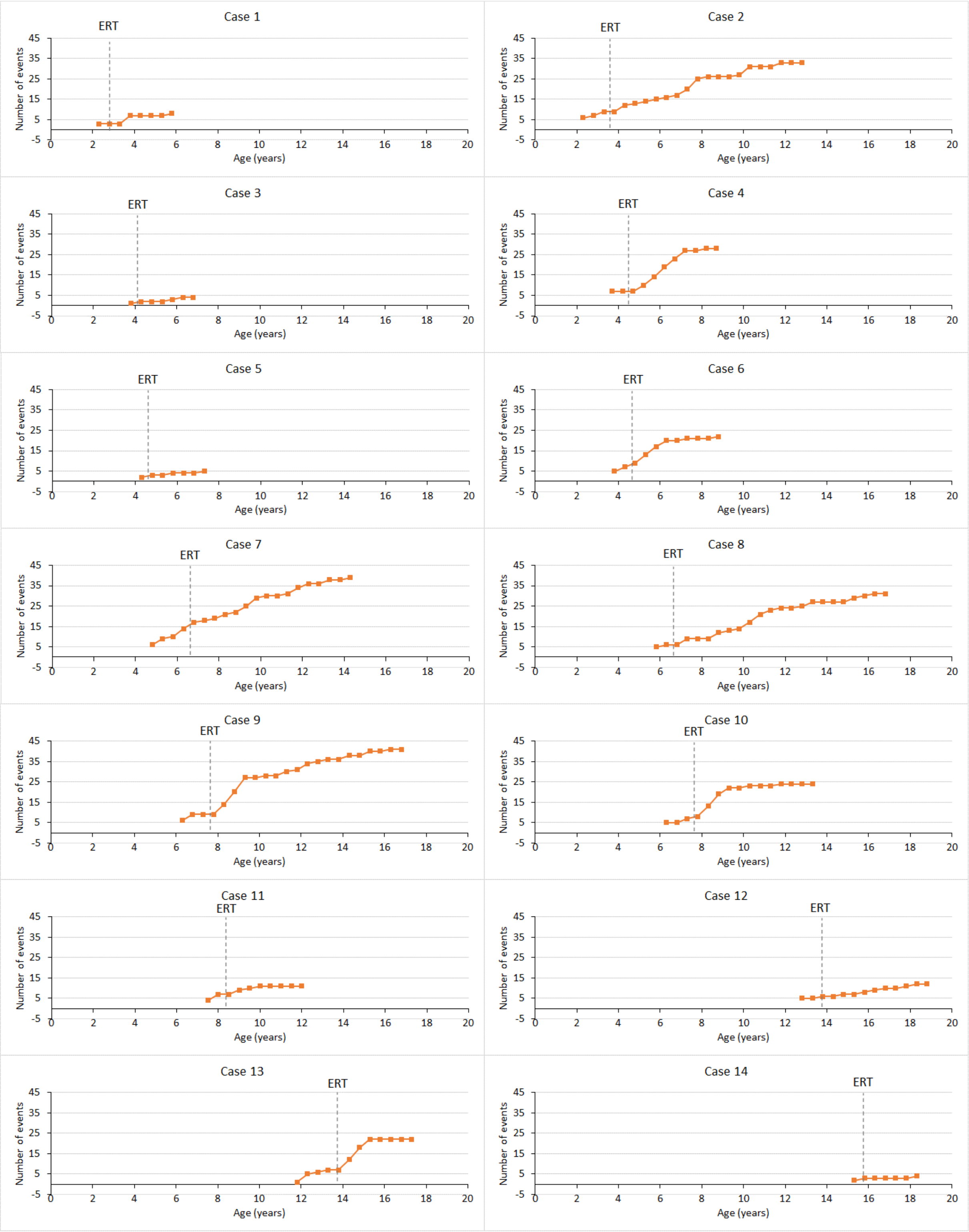

S7 Summary of event-based outcomes

Severe upper and lower respiratory tract infections

All patients, except cases 3, 5, and 14, had recurrent upper respiratory tract infections (ranging from two to four) before treatment initiation. In most cases, the frequency of infections dropped during follow-up, with only cases 5 and 14 reporting infections in the last 6 months of follow-up.

Ophthalmologic

Three patients had eyeglasses before ERT was started (cases 3, 4, 11); the remaining patients, except cases 5 and 14, started wearing eyeglasses during follow-up. Case 13 had eye pain and started taking glaucoma medication within the first year of ERT. Cases 1 and 11 were reported to have cataract around 3 years and 1.5 years after treatment initiation, respectively.

Respiratory

Sleep apnea (snoring) and/or requirement of O_2 therapy was reported for six cases before initiation of ERT (2, 6, 8, 10, 12 and 14). Sleep apnea was reported at least once after initiation of ERT in three out of six patients (2, 8 and 12), but was no longer reported after 0 to 1.5 years of treatment in the three others. Five patients (1, 4, 7, 9 and 13) developed sleep apnea or required O_2 therapy after initiation of treatment.

Upper airway

All patients, except case 3, had an adenotonsillectomy or adenoidectomy before initiation of ERT (Supplementary table S2). Cases 2, 7, and 8 had another adenoidectomy around 5 years after initiation of ERT. Case 9 had a tracheostomy around 1 year after treatment initiation.

Hearing

Case 4 had a cochlear implant before initiation of ERT. Five patients (cases 4, 6, 8, 10, 13) had ear tube insertions during follow-up.

Psychiatric

Seven patients (cases 3, 5, 6, 7, 9, 10, and 14) reported depression before initiation of ERT. Ten patients (cases 1, 2, 4, 6, 7, 8, 9, 10, 11, and 13) had periods of depression during follow-up.

Sleep

Ten patients (cases 2, 4, 5, 6, 7, 8, 9, 10, 11, 12) reported reduced quality of sleep (including frequent awakenings/interrupted sleep, sweating, and daytime sleeping) before ERT was initiated. Sleep problems persisted during follow-up in cases 6, 7, 9, but largely resolved in the other patients.

Cardiac

At baseline, case 13 was treated with an angiotensin-conversion-enzyme (ACE) inhibitor and case 2 was diagnosed with ventricular extrasystole. Eight patients were received ACE inhibitors (cases 2, 4, 5, 7, 9, 10, 11 and 13) during follow-up. Case 4 was diagnosed with minimal pericardial effusion in the first year after treatment initiation.

Mobility

No mobility events were reported before initiation of ERT. Case 7 started using a walker around 3.5 to 4 years after initiation of ERT; case 9 became immobile after around 1.5 years of treatment. Botox injections every 6 months were started to treat spasticity in cases 1 and 11 shortly after initiation of ERT.

Abdominal

Case 13 had an umbilical hernia repair before starting ERT. None of the other patients developed umbilical hernias during follow-up.

Pain

Cases 7 and 9 reported leg pain before ERT was started, and intermittently during follow-up. After treatment initiation, case 2 intermittently reported chest pain, case 4 started reporting elbow pain shortly after treatment initiation up to 3 years follow-up, case 8 reported vertebral

pain in the first year on ERT and again after 5 years of treatment until last follow-up, and case 9 reported headaches in the first year of ERT. Case 13 reported eye pain in the first year of treatment.

Neurological

Case 9 developed spinal cord compression in the first year after treatment initiation. Case 4 was diagnosed with carpal tunnel syndrome in the first year after treatment initiation.
